# Editorial: Development of functional bacteria and its application in silage processing

**DOI:** 10.3389/fmicb.2025.1625264

**Published:** 2025-05-28

**Authors:** Jie Zhao, Liwen He, Huili Pang

**Affiliations:** ^1^Institute of Ensiling and Processing of Grass, College of Agro-Grassland Science, Nanjing Agricultural University, Nanjing, China; ^2^State Key Laboratory of Animal Nutrition and Feeding (SKLANF), College of Animal Science and Technology, China Agricultural University, Beijing, China; ^3^Henan Key Laboratory of Ion Beam Bioengineering, School of Agricultural Sciences, Zhengzhou University, Zhengzhou, China

**Keywords:** microbial inoculant, functional bacteria, bioactive substance, silage fermentation, feeding value

## Introduction

Ensiling preserves forage for livestock, but its success depends on the native microbial community, forage quality, and environmental conditions. Functional bacterial inoculants—strains selected for desirable traits—offer a way to steer silage fermentation toward optimal outcomes. As noted in the Research Topic background, introducing such bacteria can “manipulate and optimize the fermentation dynamics,” leading to faster lactic-acid production, lower pH, and better nutrient preservation. Frontiers solicited contributions on isolating novel silage bacteria and improving silage fermentation with these microbes, under the broad goal of enhancing feed quality and stability.

## Application of *Lactobacillus* in silage fermentation

Tang et al. found that *Lactobacillus rhamnosus*-inoculated silages accumulated the most lactic acid (92 g/kg DM vs. 57–70 for other treatments) and the least butyric acid, indicating superior fermentation. Across crops and timepoints, inoculated silages showed suppressed spoilage bacteria during aerobic exposure: for example, harmful Stenotrophomonas and Providencia were lower in LAB-treated silages than in controls. These results highlight that selecting the right *Lactobacillus* strain can markedly improve silage stability and quality under challenging conditions.

## Synergistic or co-culture strategies

Chen et al. applied seven treatments (no additive or *L. plantarum*/*Bacillus licheniformis* at 10^5^-10^7^ CFU/g) and used 16S sequencing to track silage quality. All inoculated silages had significantly lower pH and ammonia-N than controls, and higher water-soluble carbohydrate levels. Notably, the highest *L. plantarum* dose (10^7^ CFU/g) maximized true protein content and reduced fiber fractions. Both *L. plantarum* and *B. licheniformis* also reshaped the microbiome by reducing spoilage taxa (e.g., *Sediminibacterium, Nitrospira*). Overall, this study shows dose-dependent effects of combined LAB and *Bacillus* inoculants on improving silage nutrition and suppressing undesirable microbes.

Wan et al. first isolated a cellulolytic *B. methylotrophicus* strain BM2-4 and optimized its lyophilization. Then they encapsulated *L. plantarum* (to delay its acidification) so that BM2-4 could act on cellulose first. Co-inoculating embedded *L. plantarum* with lyophilized BM2-4 (at a 2:1 ratio) significantly increased cellulose degradation (3.8% more degraded) and crude protein content (3.7% higher) compared to control. Importantly, this strategy did not lower silage pH, indicating that the slow-release LAB did not outcompete the cellulose degrader too quickly. Wan et al. thus demonstrate that engineering inoculation timing can harness synergy: delaying LAB activity gives cellulolytic partners time to improve silage fiber utilization.

Jin et al. assessed the effectiveness of single and mixed inoculants in high-protein soy silage. They found that all inoculations (*L. plantarum, L. plantarum*+*B. subtilis*, or *LP*+*Saccharomyces cerevisiae*) lowered bacterial richness and increased fungal diversity after 60 days. *L. plantarum* (alone or with co-inoculants) yielded higher lactic and acetic acids and lower pH and propionate than CK. The amino acid composition was largely unaffected, but silages inoculated with *L. plantarum* (especially *L. plantarum*+*B. subtilis*) achieved significantly higher relative feed values (e.g., 177.9 vs. ~120 for CK; *p* < 0.05). Thus, combining *L. plantarum* with other microbes can enhance fermentation and preserve protein content in legume silages.

## Microbial community dynamics and functional analysis

By using plate counting and 16S sequencing, Huang et al. found that lactic acid bacteria (LAB) and aerobic bacteria peaked at the flowering stage, yeast peaked at the milk stage, and molds peaked at full ripening. The overall epiphytic community was dominated by Proteobacteria, but the dominant genera shifted: *Pantoea, Acinetobacter* and *Pseudomonas* were most abundant at flowering, whereas *Stenotrophomonas* and *Sphingobacterium* increased at milky ripeness. This study highlights that the natural epiphytic flora—the microbial “starting inoculum” on the crop—changes dramatically with plant age, implying that silage inoculation strategies might need to be stage-specific.

Xie et al. analyzed the intestinal bacterial community structure from wood- and soil-feeding termites and discovered microbes that adept at breaking down lignocellulose. Their shotgun metagenomes revealed 26 major bacterial phyla: wood-feeding *Microcerotermes* guts were dominated by Spirochaetes (~55%), whereas soil-feeding *Pericapritermes nitobei* guts were ~95% Firmicutes. Consistently, microbial diversity was much higher in the wood-feeders. Functional prediction (Tax4Fun) showed that carbohydrate metabolism pathways predominated in both termite groups, confirming that these gut consortia are specialized for cellulose and complex polysaccharide degradation. This comparative analysis suggests that exotic microbiomes (like termite symbionts) harbor potent lignocellulolytic functions that could inspire novel silage inoculants.

Guo et al. inoculated chopped mulberry with *L. plantarum, Pediococcus pentosaceus*, and/or *Streptococcus bovis* and analyzed the chemical characteristics, antioxidant capacity, bacterial community, and metabolite composition of mulberry silage. All LAB-inoculated silages had higher dry matter retention and lower pH than controls, with combined inocula yielding the lowest ammonia-N. Antioxidant measures (FRAP, ABTS, DPPH) were significantly higher in inoculated silages, especially *L. plantarum*+*P. pentosaceus*+*S. bovis*, indicating enhanced bioactive compounds. Sequencing showed that inoculation sharply increased the abundance of *Lactiplantibacillus* and *Pediococcus* in respective treatments, restructuring the microbial community. Metabolomic profiling revealed that silages with *L. plantarum*+*P. pentosaceus* accumulated flavonoids (e.g., eriodictyol) correlated with antioxidant activity. This study is one of the first to link specific LAB inoculants with elevated silage antioxidants via combined 16S and metabolomic analyses, demonstrating that functional bacteria can enrich silage with health-promoting compounds.

In summary, these seven articles collectively highlight multiple approaches to leveraging functional bacteria for silage: from single-strain LAB inoculation to multi-species consortia and novel delivery strategies. They also underscore the importance of understanding microbial community shifts—whether naturally occurring (as in crop epiphytes) or isolated (through inoculation)—and linking these to fermentation outcomes and forage chemistry. Future perspectives: despite this progress, certain gaps remain. Many studies were short-term or lab-scale; future work should test the long-term stability and practical performance of novel strains or consortia under variable conditions. The interactive mechanisms among co-inoculated microbes need deeper exploration—for instance, metagenomic and metabolomic methods should be extended to elucidate precisely how functional bacteria alter silage biochemical pathways and microbial networks over time. In the future, synthetic microbiology and precision fermentation might enable tailored inoculant cocktails optimized for specific crops or silage goals. Addressing these challenges will require multidisciplinary efforts but promises to deliver more reliable, nutritious silages and more efficient livestock production.

